# Assessing how individuals conceptualize numeric pain ratings: validity and reliability of the Pain Schema Inventory (PSI–6) Short Form

**DOI:** 10.3389/fpain.2024.1415635

**Published:** 2024-08-05

**Authors:** Robert C. Wiederien, Dan Wang, Laura A. Frey-Law

**Affiliations:** Department of Physical Therapy and Rehabilitation Science, Carver College of Medicine, University of Iowa, Iowa City, IA, United States

**Keywords:** pain rating, questionnaire, psychometric property, Bland-Altman, pain schema

## Abstract

**Background:**

While numeric scales to represent pain intensity have been well validated, individuals use various conceptualizations when assigning a number to pain intensity, referred to as pain rating schema. The 18-item Pain Schema Inventory (PSI-18) quantifies pain rating schema by asking for numeric values for multiple mild, moderate or severe pain conditions. This study aimed to assess the validity and reliability of a shortened form of the PSI, using only 6 items (PSI-6).

**Methods:**

A secondary analysis was performed on two existing datasets. The first (*n* = 641) involved a community-based population that completed the PSI-18. The second (*n* = 182) included participants with chronic pain who completed the PSI-6 twice, one week apart. We assessed face validity, convergent validity, offset biases, test-retest reliability, and internal consistency of the PSI-6 compared to the PSI-18.

**Results:**

Both the PSI-18 and PSI-6 demonstrated excellent face validity. The PSI-6 demonstrated excellent convergent validity relative to the PSI-18, with correlations from r = 0.88 to 0.92. Bland-Altman plots revealed offset biases near zero (< 0.22 on 0–10 scale) across all categories of mild, moderate, severe and average pain. Internal consistency was excellent, with Cronbach's Alpha = 0.91 and 0.80, for PSI-18 and PSI-6 respectively. Test-retest reliability of the PSI-6 was high with correlations from r = 0.70–0.76.

**Conclusion:**

The PSI-6 is a valid and reliable tool to assess pain rating schema with reduced subject burden, to better interpret individuals’ pain ratings and adjust for inter-individual variability.

## 1 Introduction

Pain is the most widely reported symptom in the medical literature, with an estimated 56% of American adults reporting some pain in the previous 3 months, and an estimated 20% reporting chronic pain (1, 2). Although pain can vary widely, the International Association for the Study of Pain encompasses this range by defining it as, “An unpleasant sensory and emotional experience associated with, or resembling that associated with, actual or potential tissue damage” (3). Multiple methods have been used to assess the subjective nature of pain including unidimensional numerical intensity ratings (4–8) and multidimensional assessments (9, 10). While multidimensional approaches provide unique insight, unidimensional pain ratings are widely used for their ease, compliance, and responsiveness (7, 8).

The psychometric validity of pain rating assessment has been well documented (4–6, 8, 11–13). However, underlying pain rating schemas can vary across individuals, i.e., the conceptual frameworks that individuals utilize when transforming pain to a number (14). While moderate pain is consistently rated numerically higher than mild pain, and severe pain higher than moderate pain, the actual numerical pain ratings for each level (i.e., mild, moderate, or severe pain) can greatly differ between individuals. This may add challenges when interpreting either clinical or research pain ratings, as what is a “3” to one person may be a “4” to another, and so on. Identifying these differences in underlying pain rating schema may improve the interpretation of individuals’ numeric pain ratings.

More broadly, generalized pain schemas have been described as pain-related information processing biases or internal sources of information (15) as well as “general beliefs, appraisal and expectations about pain” (16). Although challenging to assess, generalized pain schemas, i.e., the internal frameworks that help interpret anticipated or actual painful experiences, have been evaluated using paired word choices (e.g., “excruciating”—“relieving”), sentence completion (e.g., Sentence Completion Test), and both explicit means of assessment, as well as implicit assessment using the Implicit Association Test (IAT) (17, 18). These general assessments of pain schema often consider the multidimensional nature of pain. Some experts may even consider assessments of pain catastrophizing or pain-related fear of movement to be forms, or components, of generalized pain schema, i.e., assessing beliefs, appraisals or expectations about pain (19, 20).

One specific component of the generalized pain schema is pain rating schema, which considers how one represents pain intensity numerically (14). The evaluation of pain rating schema is distinct from the explicit and implicit methods used to assess more generalized interpretive biases, explicitly attempting to better understand how individuals conceptualize pain intensity. While numerical ratings of pain as a sensory and emotional experience have become routine both in the clinic and in research settings, relatively few attempts have been made to quantify the schema underlying these numerical assignments (8).

Numerous studies have examined individual differences in pain sensitivity, frequently utilizing numerical pain ratings as the primary independent or dependent variable. That is, they evaluate for differences in pain ratings for a given, standardized noxious input, or conversely identifying variation in the noxious input needed to achieve a particular pain intensity (7, 21, 22). Either way, the underlying pain rating schema is inherently a core component of how individuals conceptualize their pain numerically. However, this schema is rarely considered or adjusted for, which can lead to inaccurate or misleading inferences when comparing pain ratings between individuals. Similarly, clinical pain science often relies on self-reported pain intensity to study relationships with pain biomarkers, including imaging, psychosocial, and “omics” variables (23). Further, minimum pain rating intensities are frequently used as a criterion for inclusion in clinical trials (24). Yet, few clinical studies consider adjusting for individual variation in the use of numerical pain scales, i.e., pain rating schema.

In one cross-sectional study, generalized pain rating schema were identified as average cut-off values for mild, moderate, and severe pain based on corresponding functional interference scores (25). While it was not able to identify individual-specific pain schema, cut-off values were related to pain catastrophizing, with no sex differences (25). In another study, individuals were asked to rate their anticipated pain ratings for mild, moderate, and severe pain for five different common acute pain conditions (headache, toothache, joint pain, delayed onset muscle soreness, and burns) and pain in general (14). While not originally named, we refer to this survey as the Pain Schema Inventory (PSI). In a mixed cohort of community-dwelling adults, with and without pain at the time of completion, three pain schema clusters emerged (14). One cluster (“Low rating subgroup”) predominantly utilized the lower portion of the 0–10 scale (i.e., mean mild to severe pain range: −1.5 to −5.5 out of 10). A second cluster (“High rating subgroup”) predominantly utilized the upper portion of the scale (mild to severe range: −2.5 to −7.5 out of 10). While a third cluster (“Low/high rating subgroup”) utilized the fullest range of the 0–10 scale, (mild to severe range: −1.5 to −7.5). The relative consistency in pain ratings across hypothetical pain conditions supports the presence of pain rating schema. However, a recent study identified that individuals sometimes choose to over- or under- report their numeric pain ratings for specific situations or motivations such as not being taken seriously or to meet others’ expectations (26). Even in these cases, individuals must rely on an underlying schema to conceptualize their actual pain.

More recently, Wang, et al. (2023) applied a shortened version of the PSI survey for use as a covariate to adjust for differences in pain-rating schema when evaluating the relationships between multisensory sensitivity and number of chronic pain conditions (27). The shortened version, which assessed only headache and joint pain, was used to reduce subject burden, from 18 items to 6 items. While the original scale has shown good test-retest repeatability, high face validity and high internal consistency (14), whether the psychometric properties of the shortened form are similar was unreported. Thus, the purpose of this study was to examine the validity and reliability of a short form of the Pain Schema Inventory (PSI-6) using existing datasets.

## 2 Materials and methods

A retrospective secondary analysis was conducted using two datasets (Wang, et al, 2022; Wang, et al, 2023) to assess multiple psychometric properties of the PSI-6 (27, 28).

### 2.1 Participants

All participants completed multiple surveys in the original studies using the Research Electronic Data Capture interface (REDCap) (27, 28). Both studies were approved by the University of Iowa Institutional Review Board using a waiver of consent. The first dataset, Cohort 1, included 641 participants from a community-based population with and without pain who completed 18 items of the PSI (28). The second dataset, Cohort 2, included participants with chronic pain (*n* = 182) who completed the PSI-6 twice, one week apart (27). There was no overlap in participants from each cohort.

### 2.2 Pain Schema Inventory (PSI-18 and PSI-6)

The original study evaluating numerical pain rating schema asked participants to rate what they would perceive as mild, moderate and severe pain (3 categories) for 6 pain conditions: headache, muscle soreness, toothache, joint injury, burn, and pain “in general” (we refer to these as the PSI-18) (14). Pain threshold and tolerance were also originally explored (12 items, 30 total) but threshold and tolerance may have additional implications beyond numerical pain assignment, they have not typically been included in subsequent assessments. The PSI-6 includes the numeric ratings for mild, moderate and severe pain for only 2 of the conditions (headache and joint injury) (27, 28). The first dataset (Cohort 1) asked participants to complete the original 18 mild, moderate, and severe items, which allowed us to extract both the PSI-18 and PSI-6 for comparison, whereas the second dataset (Cohort 2) only assessed the PSI-6, but at two time points. We also explored using only the 3 PSI pain “in general” items (PSI-3) in Cohort 1. The evaluation of a subset extracted from the full instrument has been used previously, e.g., the SF-12 and SF-36 (29, 30).

In the original study, the PSI was assessed using a paper interface with a 10 cm visual analog scale (VAS) where 0 cm = no pain and 10 cm = worst pain imaginable (14). The current datasets (Cohorts 1 and 2), however, used an electronic survey interface where the PSI items were assessed with a 0–10 numeric rating scale (NRS) with the option to use whole or half numbers (0.5 increments). The PSI-18, PSI-6 and PSI-3 items and scoring are provided in the Appendix.

### 2.3 Statistical analyses

Summary statistics were computed for all primary measures (mean mild pain, moderate pain, severe pain, and overall average pain rating across all three pain levels) for both the PSI-18 and PSI-6.

#### 2.3.1 Validity

*Validity*, defined as the extent to which a concept is accurately measured (31), can be assessed in multiple ways. We assessed both *face validity*, “the extent that an instrument measures the concept intended” (31) and *convergent validity*, a subset of criterion validity, which is typically assessed as how well one instrument measures similar constructs as another (31, 32). Both forms of validity were first assessed using a two-way repeated measures analysis of variance (ANOVA) for the first dataset (Cohort 1). The repeated factors included: category (mild, moderate, and severe) and form (PSI-18 and PSI-6). Additional covariates included sex (male = 0, female = 1) and pain status (no = 0, yes = 1). The Huynh-Feldt correction for non-sphericity was applied as needed. Effect sizes were computed as partial eta squared (*η*^2^) values and interpreted as: negligible (*η*^2 ^< 0.01), small (0.01 ≤ *η*^2^ < 0.06), medium (0.06 ≤ *η*^2^ < 0.14), and large (*η*^2^ ≥ 0.14) (33).

*Post hoc* tests were performed as appropriate using paired t-tests with Bonferroni corrections. Simple effect sizes (Cohen's d) were also computed for each pair. One would expect mild pain to be numerically less than moderate or severe pain for the PSI-18 and the PSI-6. That is, intensity ratings should increase from mild to moderate to severe, with severe intensities being the highest as an indicator of face validity.

Convergent validity was assessed first by comparing pain ratings between the two forms, PSI-18 and PSI-6 using the two-way repeated measures ANOVA described above. In addition, Pearson's correlation coefficients for each mild, moderate, and severe pain category, as well as the average pain ratings between the PSI-6 and both the original PSI-18 and the remaining items from the PSI-18 omitted from the PSI-6 (i.e., the 12 items not included in the PSI-6) were performed. These approaches enable the evaluation of the consistency between the PSI-6 and its original version (primary outcome) as well as with the portion of the full form without duplication, thereby minimizing over-estimating convergence due to shared items (secondary outcome). We operationally defined acceptable convergent validity as r ≥ 0.7 (32), anticipating convergence with the original full PSI-18 to be higher than convergence with the items excluded from the PSI-6. We also assessed the correlations between the PSI-3 compared to the PSI-18 to explore if a further shortened version might be comparable, or an alternative, to the PSI-6.

Finally, we evaluated differences between PSI-18 and PSI-6 forms using Bland-Altman analyses, again using the first dataset (34, 35). Bland and Altman proposed that mean pairwise differences should be evaluated relative to mean pairwise values to evaluate for overall bias in the magnitude of the new scale. Accordingly, we plotted the differences (PSI-6—PSI-18) on the y-axes relative to the mean of the PSI-6 and PSI-18 scores on the x-axis for each pain severity category (mild, moderate, and severe) as well as the overall average pain rating across all categories. The mean difference (offset bias) and 95 percent limits of agreement for each Bland-Altman plot were computed and compared to the minimal clinically important difference (MCID) of pain, 2.3 on a 0–10 scale (36), as a reasonable estimate for comparison.

#### 2.3.2 Reliability

*Reliability* is the consistency of a measure, which too can be assessed in multiple ways (31). *Internal consistency* is described as the extent to which items within an instrument are measuring the same underlying construct (31). We assessed internal consistency for both the PSI-18 and PSI-6 for Cohort 1 and the PSI-6 for Cohort 2 (first visit) with Cronbach's alpha. Optimal internal consistency has been previously reported with good or acceptable values between 0.70 and 0.95 (32), as overly high internal consistency (r > 0.95) may signify unnecessary redundant items within an instrument.

*Test-retest reliability* assesses the consistency of a measure when given to the same participants more than once under similar circumstances (31). Pearson correlation coefficients were computed between two assessments of the PSI-6, completed one week apart, using the second dataset (Cohort 2). Consistent with convergent validity, we operationally defined acceptable reliability as r ≥ 0.70 (32).

Significance levels were set at alpha ≤0.05 for all analyses, and statistical analyses were performed using IBM SPSS Statistics for Windows, version 28 (IBM Corp., Armonk, N.Y., USA).

## 3 Results

Details on Cohorts 1 and 2 including sex, age, race, ethnicity, educational level, and current as well as worst pain over the past 7 days are summarized in Table 1 (27, 28).

**Table 1:** Participant characteristics reported as mean (SD) or *n* (%) as appropriate.

Characteristic	Cohort 1 (*n* = 641)	Cohort 2 (*n* = 182)
Age (years)	48.4 (20.4)	26.2 (12.4)
Sex		
Male	188 (29.3%)	28 (15.4%)
Female	452 (70.5%)	154 (84.6%)
No answer	1 (0.2%)	0 (0%)
Race		
American Indian/Alaska Native	4 (0.6%)	2 (1.1%)
Asian	30 (4.7%)	11 (6.0%)
Black or African American	7 (1.1%)	5 (2.7%)
Native Hawaiian/Pacific Islander	2 (0.3%)	2 (1.1%)
White or Caucasian	597 (93.1%)	163 (89.6%)
Other	7 (1.1%)	3 (1.6%)
Unknown/no answer	4 (0.6%)	4 (2.2%)
Ethnicity		
Hispanic or Latino	17 (2.7%)	17 (9.3%)
Not Hispanic or Latino	594 (92.7%)	162 (89.0%)
Unknown/no answer	30 (4.7%)	3 (1.6%)
Education (highest level attained)		
Grade school	12 (1.9%)	0 (0.0%)
High school/GED	16 (2.5%)	34 (18.7%)
Some college or trade school	84 (13.1%)	66 (36.3%)
College	257 (40.1%)	51 (28.0%)
Post-graduate or doctoral	268 (41.8%)	31 (17.0%)
No answer	4 (0.6%)	0 (0.0%)
Worst pain last 7 days (0–10)	3.4 (2.4)	3.8 (2.3)
Pain status at time of survey		
Yes (*N*, %)	399 (62.2%)	112 (61.5%)
If yes, intensity (0–10)	2.4 (2.3)	2.3 (1.9)

### 3.1 Validity

**Figure 1:**
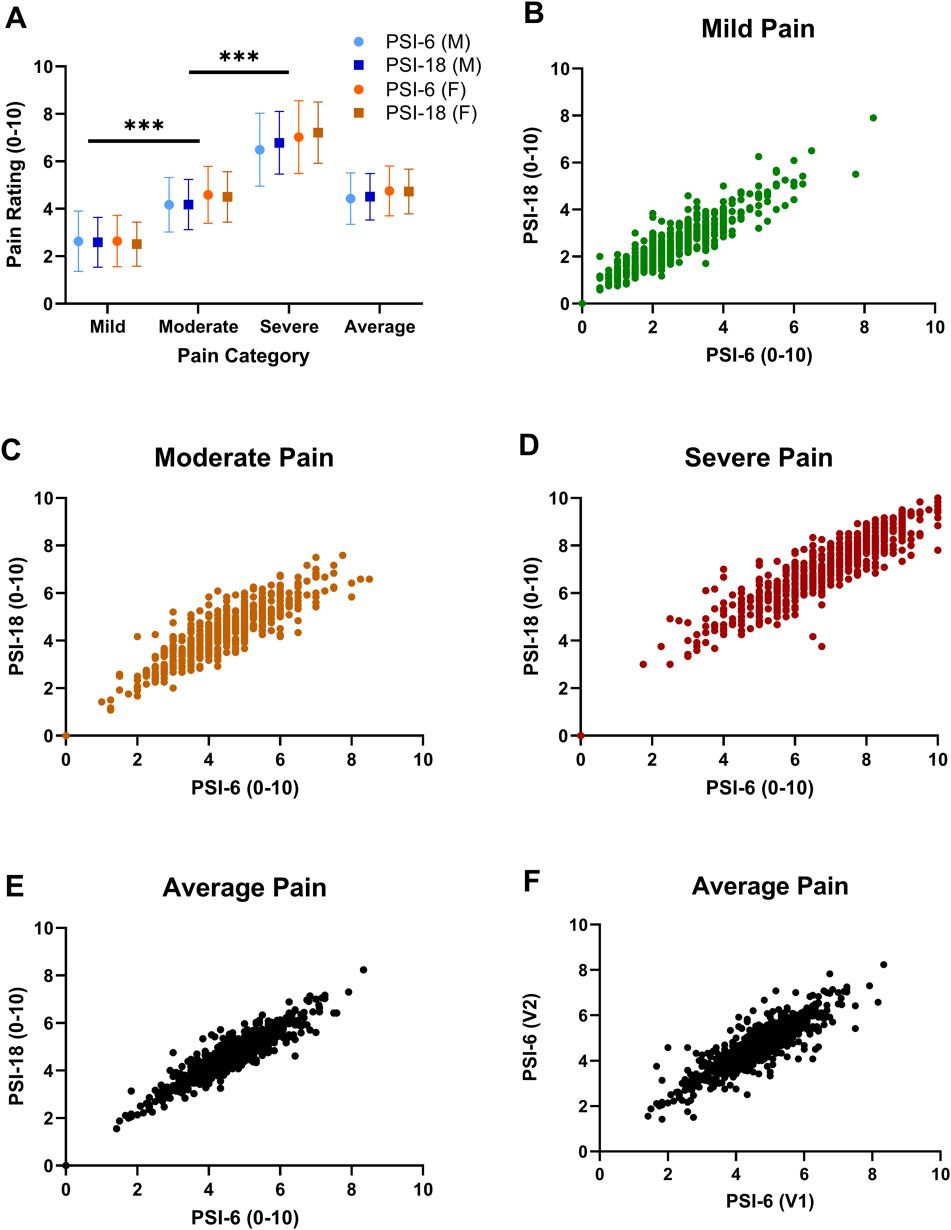
Mean (SD) pain ratings are shown in panel (**A**) for each pain category (mild, moderate, and severe pain) and the overall average pain rating across all items with PSI-6 (circles) and the PSI-18 (squares) and males in blue and females in orange. Face validity is demonstrated as mild pain ratings were significantly less than moderate, and moderate significantly less than severe pain. Significant sex differences were also noted, but with very small overall effect sizes (*p* = 0.006, *η*^2^ = 0.012) and inconsistent across pain categories. Significance signified by ***p < 0.001. Panels (**B–E**) show scatterplots of pain ratings using PSI-6 vs. PSI-18 from cohort 1 to evaluate construct validity, where B) mild pain (green), (**C**) moderate pain (orange), (**D**) severe pain (red), and (**E**) overall average pain (black). In panel (**F**) the overall test-retest stability of the PSI-6 is shown as a scatterplot of average pain ratings obtained from visit 1 (V1) vs. visit 2 (V2) for cohort 2.

Both the PSI-18 and PSI-6 demonstrated good face validity (see Figure 1A), as the numerical pain ratings were consistently lowest for mild pain, higher for moderate pain, and highest for the severe pain category (*p* < 0.0001), with an overall very large effect size (*η*^2^ = 0.853). While differences between the original (PSI-18) and short form (PSI-6) were observed (*p* = 0.048, see Table 2), the overall effect size due to form was negligible (*η*^2^ = 0.006), evidence for good construct validity. While several two-way interactions were statistically significant, including pain category (mild, moderate, severe) by form (PSI-18, PSI-6); pain category by sex; and form by sex (see Table 2), the effect sizes were again typically small or negligible (*η*^2^ ≤ 0.02) for all but category by form which was moderate (*η*^2^ = 0.082). These effects were dwarfed by the differences observed between mild, moderate, and severe pain.

**Table 2:** Statistical analysis results.

Bivariate correlations (cohort 1)	PSI-6 vs. PSI-18	PSI-6 vs. 12 omitted PSI-18 items	PSI-3 vs. PSI-18
Mild (r)	.90***	.75***	.70***
Moderate (r)	.88***	.73***	.70***
Severe (r)	.90***	.90***	.68***
Average (r)	.92***	.86***	.75***
Internal consistency	PSI-6	PSI-18	
Cohort 1 (α)	.80	.91	
Cohort 2 (α)	.88	–	
Test-retest correlation (cohort 2)	PSI-6	–	
Mild (r)	.70***	–	
Moderate (r)	.70***	–	
Severe (r)	.75***	–	
Average (r)	.76***	–	
ANOVA (cohort 1)	F-statistic	*p*-value	Effect size (*η*^2^)
Pain category (mild, moderate, severe)	3,659.67	<.0001***	.853
Form (PSI-18 vs. PSI-6)	3.92	.048*	.006
Sex (M/F)	7.46	.006**	.012
Pain Status (Y/N)	2.46	.12	.004
Sex X pain status	1.98	.16	.003
Pain category X form	56.45	<.001***	.082
Pain category X sex	12.94	<.001***	.020
Form X sex	6.21	.013*	.010
Pain category X pain status	0.34	.670	.001
Form X pain status	0.11	.742	.000
Pain category X form X sex	0.06	.940	.000
Pain category X form X sex X pain status	1.53	.218	.002

Significance signified as follows:

* *p* < .05.

** *p* < .01.

*** *p* < 0.001.

Significant main effects of sex were also observed, but with a small effect size (*η*^2^ = 0.012, see also Figure 1A). *post hoc* paired t-tests revealed significant (*p* < 0.02) but small and inconsistent effect sizes for sex differences at each pain level (Cohen's d): mild (d = 0.10); moderate (d = 0.05); severe (d = −0.15). There were no significant differences in pain ratings by pain status (yes/no) for main (*p* = 0.12) or any interaction effects (*p* > 0.22).

Excellent convergent validity of the PSI-6 was demonstrated by high correlations with the PSI-18 for mild, moderate and severe pain categories (r = 0.88–0.90; *p* < 0.001), as well as for the overall average pain rating (r = 0.92; *p* < 0.001) (See Table 2). This means the PSI-6 explained 77%–85% of the variance observed in the full PSI-18 with only one-third the items. Similarly, the PSI-6 also demonstrated good to excellent convergent validity when compared to the 12 PSI items omitted from the PSI-6, r = 0.73–0.90 (*p* < 0.001) for mild, moderate, severe, and average pain (Table 2). While pain “in general” as a single item showed good correlations with the PSI-18 (*p* = 0.68–0.75; *p* < .001), they were consistently lower than the for the PSI-6, explaining 46%–56% of the PSI-18 variance.

**Figure 2:**
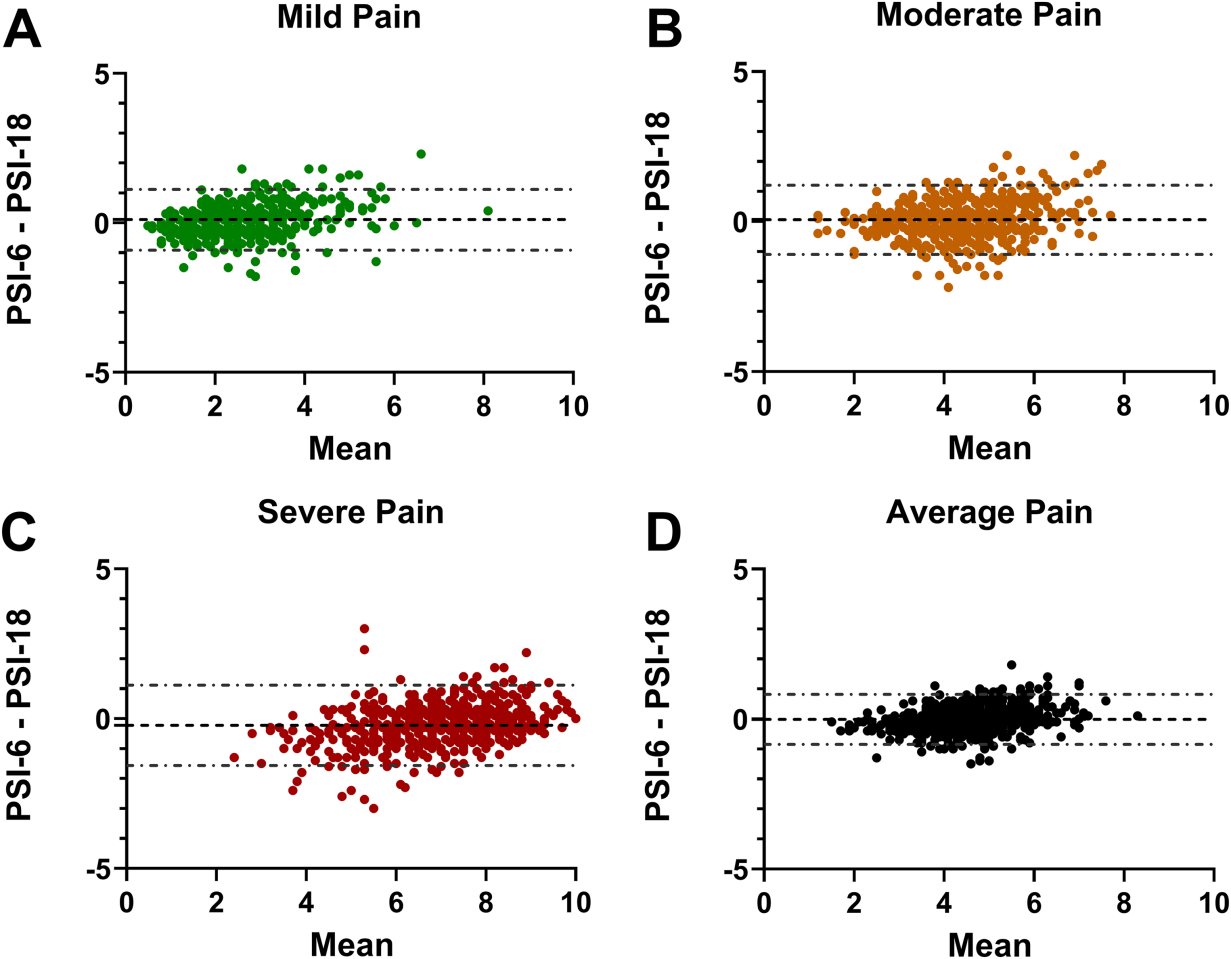
Bland-Altman plots showing the PSI-6 minus the PSI-18 difference on the y-axes and the mean PSI-6 and PSI-18 on the x-axis. The mean offset bias between the two is shown with a dashed line, and 95% limits of agreement shown with dash-dot lines for (**A**) mild pain; (**B**) moderate pain; (**C**) severe pain; and (**D**) the average pain across all pain levels. Note that the 95% limits of agreement for all four plots are notably less than the reference MCID (2.3) used for this study, with only a few individuals with differences exceeding the MCID.

The Bland-Altman analyses demonstrated mean offset biases close to zero across all pain levels: mild (0.11), moderate (0.06), and severe (−0.22) as well as the overall average (−0.01) (Figures 2A–D). The 95% limits of agreement were all well less than the MCID cut-off of 2.3: with ± limits of 1.01 (mild), 1.16 (moderate), 1.34 (severe) and 0.84 (average). In each plot, only a few select individuals showed differences that exceeded the 2.3 MCID.

### 3.2 Reliability

Internal consistency of the PSI-18 and PSI-6, using the first dataset (Cohort 1), were good to excellent with Cronbach's Alpha = 0.91 and 0.80, respectively (Table 2). Similarly, in Cohort 2 Cronbach's Alpha was 0.88 for PSI-6. Test-retest reliability of the PSI-6 based on mild, moderate, and severe pain Pearson's correlation coefficients was good to high, with values ranging from 0.70 to 0.76, *p* < 0.001 (Table 2).

## 4 Discussion

The assessment of pain rating schema using the PSI-6 showed excellent face and convergent validity, high test-retest reliability and internal consistency. Our results indicated a high degree of consistency and agreement between the PSI-18 and the PSI-6. While some variability was noted using the Bland-Altman analyses, and the very small differences observed were statistically significant with the large sample size, no substantial or meaningful offset was observed. Collectively, these assessments suggest the brief, PSI-6 is a valid and reliable tool to assess pain rating schema with low respondent burden.

Numerous efforts have been made to better understand an individual's pain experience, from simple numeric pain ratings to fuller assessments including pain descriptors such as the McGill Pain Questionnaire (9). Indeed, studies comparing others’ perception of an individual's pain experience demonstrate often poor estimation of pain intensity, that can be influenced by the surrogate raters’ pain or health status (37–39). Imaging and other biomarker studies have long been searching for the elusive objective measure of pain (40, 41), yet pain by definition is a sensory and emotional experience (3). Thus, we will always need to rely, at least to some degree, on asking individuals to communicate in some way their perception of their pain. A benefit of utilizing additional pain assessment methods or instruments beyond a NRS or VAS may be a deeper understanding and insight into the individual's pain experience (42).

Characterizing one's pain rating schema may aid in interpreting their ratings of pain intensity. Multiple studies have shown that there is a high degree of variability in how different individuals report their pain (7, 14, 16, 43), including intentional over- or under-reporting of pain for a variety of reasons (26). Patients with pain may have information-processing biases that can be at least partially explained by their pain rating schemas (14). Despite the widespread use of numerical scales to assess pain, there are very limited tools available to assess the construct of pain rating schema (42). The PSI-6 retains the ability of the PSI-18 to assess for an overall tendency to rate pain intensity across all levels (mild through severe) using a single mean score, or evaluate levels separately (e.g., mild vs. moderate vs. severe). Previous work demonstrated individual differences in pain rating schema, suggesting that the 0–10 rating that one individual perceives as mild may be another's moderate pain (14). Thus, differences in pain rating schema between individuals, if unrecognized, may lead to misunderstanding or misinterpretation of the others’ pain experience. For example, clinicians may undervalue patient report of pain intensity, for a variety of reasons, including implicit biases (26, 39, 44). Certainly, concerns of not being believed or having their pain taken seriously by patients compounds these challenges in communicating pain ratings (44). As pain is, by definition, a subjective experience (3), the identification of underlying pain schemas is not meant to reduce subjectivity but rather improve interpretation of the personalized pain experience. While not able to fully correct for the many nuances that may influence pain ratings, this assessment may help increase awareness of looking beyond unidimensional measures of pain as individuals sometimes find assigning numeric values to their pain inherently challenging, as we have observed anecdotally in both research and clinical settings.

Assessing hypothetical pain schema vs. simply asking an individual to also categorize their pain as mild, moderate, or severe provides two different but useful paradigms. An instrument comprised of hypothetical pain conditions provides a means to estimate how any individual uses a numeric pain scale, whether they are currently in pain. In the large cohort in which pain status (presence/absence) was considered, there was no effect on pain schema. This suggests that pain does not inherently bias individuals to change their underlying schema. The use of hypothetical pain items may be particularly helpful for research applications in which assessment across the severity range may serve to reduce pain rating heterogeneity and subsequently increase the precision for identifying underlying neurophysiologic pain mechanisms [e.g., see Wang, et al. (27)]. However, in clinical applications, it may be more feasible and immediately helpful to ask patients to report both numeric pain and whether it is best identified as mild, moderate or severe, as previously recommended (14). This approach has also been used to support cohort-wide pain rating schema determination, i.e., through the identification of mild, moderate, and sever pain cut-points (25), but is unable to generate individual-specific insights on numeric pain scale use.

When the PSI was first introduced, the authors used a 0–10 cm VAS for the pain ratings, finding good to high test-retest reliability and high internal consistency (14). Using a 0–10 NRS with 0.5 increments (21-point scale), we found similarly good to excellent psychometric properties of both the PSI-18 and PSI-6. This suggests that the PSI-18 and the PSI-6 are valid using either the 10 cm VAS or a 0–10 NRS (at least with the higher precision of 0.5 increments).

While longer questionnaires may provide the ability to detect more nuanced evaluation of any construct, the benefit of short forms is reduced subject burden and potentially, higher subject satisfaction and completion rates (45, 46). For example, the Patient-Reported Outcome Measurement Information System (PROMIS) measures typically have multiple versions, including several brief (i.e., 4-, 6- or 8-item) short forms. The PSI-6 was found to be a valid and reliable tool without a substantial loss of information from the PSI-18, making it desirable for both research and clinical applications. Further reducing burden using the PSI-3, relying only on single mild, moderate, and severe pain “in general” ratings, provides a reasonable estimate of the full PSI-18 (explained 46%–56% of the variance in PSI-18), yet was substantially worse than using the PSI-6 (explained 77%–85% of the variance in PSI-18). Thus, while both may be useful, the PSI-6 appears to represent the PSI-18 more consistently.

There are several limitations of the current study. First, the PSI assessment of pain rating schema does not reduce subjectivity as both numeric pain ratings and the categorization of pain as mild, moderate, or severe are subjective. However, their comparison provides at least a contextual framework to interpret numeric pain ratings. Another limitation is that the PSI-6 was extracted from the full PSI-18 and not collected separately. However, this approach has been used previously to assess shortened versions of instruments (29, 30, 45). Additionally, the optimal choice of which 6 items to use for the PSI-6 was not evaluated, but rather an assessment of the 6 items previously used (27, 28). It may be that a different reduced set of items (and/or number of items) could result in even greater convergence with the original PSI-18, however, the use of 2 pain conditions (headache and joint pain) was superior to just the single item of pain “in general.” Another challenge is that cohort diversity was limited. There were a limited number of men in both datasets, despite the minimal sex differences, we cannot rule out that the small number of men in our data is less representative of the larger population. Similarly, the cohorts were predominantly Caucasian and from the United States, thus may not generalize to all races, ethnicities, or other cultures.

## 5 Conclusion

This secondary analysis revealed that the PSI-6 is a valid and reliable tool to assess pain rating schema. This measure may be used to help adjust for variability in pain ratings among individuals, using the overall mean pain rating or the combination of pain ratings at each pain level (mild, moderate, severe), particularly for investigations in which pain ratings are a primary study outcome. Additional research is recommended to help further our understanding of pain rating schemas, including examining for systematic biases across pain populations, or with time following the development of chronic pain.

## Data Availability

The data analyzed in this study is subject to the following licenses/restrictions. Requests to access these datasets should be directed to laura-freylaw@uiowa.edu.

## Appendix

### Pain Schema Inventory (PSI-18, PSI-6*, PSI-3†).

Please rate the following items on a 0–10 scale, with 0 being no pain and 10 being the worst pain imaginable, using half or whole numbers.

1. What would you rate as a moderate burn (e.g., from a hot stove)?
2. What would you rate as a severe joint injury (e.g., ankle sprain or arthritis)? *
3. In general, what would you rate as a mild pain? **†**
4. What would you rate as a moderate headache? *
5. What would you rate as a severe burn (e.g., from a hot stove)?
6. What would you rate as a mild toothache?
7. What would you rate as severe muscle soreness (1–2 days after activity)?
8. What would you rate as moderate muscle soreness (1–2 days after activity)?
9. What would you rate as mild muscle soreness (1–2 days after activity)?
10. In general, what would you rate as a moderate pain? **†**
11. What would you rate as a severe toothache?
12. What would you rate as a moderate joint injury (e.g., ankle sprain or arthritis)? *
13. What would you rate as a mild headache? *
14. What would you rate as a mild joint injury (e.g., ankle sprain or arthritis)? *
15. In general, what would you rate as a severe pain? **†**
16. What would you rate as a mild burn (e.g., from a hot stove)?
17. What would you rate as a severe headache? *
18. What would you rate as a moderate toothache?

Notes and Scoring Instructions:

• Differences in pain rating schema may be characterized using the overall pain schema value as a covariate to adjust for overall tendency to use higher vs. lower range of the pain scale.
• For PSI-6 or PSI-3, only include the respective 6 (*) or 3 (†) items noted (see Table A1).
• Alternate pain scales have been used, including a 10 cm visual analog scale and the Borg CR10. We recommend allowing for 0.5 increments rather than only integers if using the 0–10 NRS.

**Table A1:** Scoring of PSI.

Category	PSI-18	PSI-6	PSI-3
*Mild pain rating:* mean of respective mild pain items	=sum(#3, #6, #9, #13, #14, #16)/6	=sum(#13, #14)/2	=#3
*Moderate pain rating:* mean of respective moderate pain items	=sum(#1, #4, #8, #10, #12, #18)/6	=sum(#4, #12)/2	=#10
*Severe pain rating:* mean of respective severe pain items	=sum(#2, #5, #7, #11, #15, #17)/6	=sum(#2, #17)/2	=#15
*Overall pain rating:* mean of all items	=sum(#1 - #18)/18	=sum(#2, #4, #12, #13, #14, #17)/6	=sum(#3, #10, #15)/3

For more information on this scale, see: Frey-Law LA, Lee JE, Wittry AM, Melyon M Pain Rating Schema: Three Distinct Subgroups of Individuals Emerge When Rating Mild, Moderate, and Severe Pain. *J Pain Res* (2013) 7:13-23. Epub 20131223. doi: 10.2147/JPRS52556.
